# Dynamic Double Cross-Linked Self-Healing Polysaccharide Hydrogel Wound Dressing Based on Schiff Base and Thiol-Alkynone Reactions

**DOI:** 10.3390/ijms232213817

**Published:** 2022-11-10

**Authors:** Zhuojun Zhang, Jiasen Bu, Biyun Li, Hongyun Xuan, Yan Jin, Huihua Yuan

**Affiliations:** School of Life Sciences, Nantong University, Nantong 226019, China

**Keywords:** wound dressing, skin repair, antibacterial, hydrogel, double cross-links

## Abstract

In this study, a hydrogel composite wound dressing with antibacterial and self-healing ability was prepared using cysteine-modified carboxymethyl chitosan, sodium oxidized alginate, and but-3-yn-2-one base on Schiff base and thiol-alkynone double cross-links. The structure and properties of the hydrogel were characterized by scanning electron microscope, Fourier-transform infrared, and rheological test, followed by antibacterial and in vivo biocompatibility tests. The results showed that the hydrogel exhibited good self-healing, mechanical properties, good antibacterial effect, and in vivo biocompatibility, and can inhibit inflammation and promote skin tissue regeneration in mice. This novel self-healing hydrogel dressing has a broad application prospect in skin tissue engineering.

## 1. Introduction

The skin is the primary physicochemical barrier between the body and the environment and plays an important role in regulating body temperature and fighting microbial infections. Therefore, damage to the skin affects or even endangers human health [1]. The skin inevitably suffers from various injuries, including burns, abrasions, and lacerations, which can affect its integrity and even lead to serious health problems; infection and damage suffered during the healing process can result in chronically infected wounds and eventually lead to amputation or even death [2]. Therefore, it is of utmost importance to take measures to accelerate skin repair and perform necessary wound care following skin injury. In recent years, many skin substitutes, such as skin grafts and dermal substitutes, have been used for skin wound repair; however, these substitutes have not been widely used due to various reasons, including donor shortage and sequelae [3].

Skin wound dressings are an effective means to treat wounds. Traditional wound dressings have the disadvantages of poor antimicrobial performance, insufficient mechanical performance, and low repair efficacy. The rapidly developing field of skin tissue engineering is a new approach to address these problems, with the aim of developing novel skin substitutes for use as wound dressings [4,5]. Several materials, natural or synthetic, have been used to make wound dressings. Among these, hydrogel dressings, which can mimic the wound repair microenvironment, have attracted a lot of attention. They have adjustable chemical, physical, and biological properties, as well as strong absorption ability based on a three-dimensional network structure. The structure and function of different hydrogels play a crucial role in skin wound repair, protecting the wound from pathogens and accelerating the healing process [6,7]. As ordinary hydrogels are easily damaged by external forces, those with self-healing effects have attracted research interest [8]. The preparation of hydrogels is usually carried out by chemical bonding to form covalent or non-covalent cross-linked networks. Thus, synthesis using a simple Schiff base reaction has become one of the common methods to prepare self-healing hydrogels. Compared with other chemical bonds, the covalently cross-linked network formed by dynamic Schiff base bonds gives hydrogels a series of properties, such as pH responsiveness, reversibility, and biocompatibility, among others, and makes Schiff base bond-based hydrogels extremely promising for tissue engineering applications [9].

Natural hydrogel materials commonly used for skin repairs, such as chitosan, alginate, and hyaluronic acid, exhibit certain properties, such as biocompatibility, biodegradability, and low cytotoxicity, and researchers usually use chemical modification to functionalize polymers of o-diols to generate aldehyde groups [10,11]. Alginate, as a natural macromolecular polysaccharide, possesses hydrophilicity, excellent biocompatibility, and fluid absorption ability, making it an excellent wound dressing material [12]; its oxidation by sodium periodate reduces its molecular weight and improves biodegradability, making it even more appealing. Chitosan is a more common natural polysaccharide, which exhibits antimicrobial properties due to its surface positively charged amino groups that adsorb negatively charged phosphopeptide or lipopolysaccharide molecules from bacterial cell walls [13]. In addition, chitosan has been widely used in the preparation of hydrogels because of its low cost, bioactivity, and biocompatibility. It can also react with aldehyde-based alginate to form Schiff base bonds, forming hydrogels with self-healing properties.

However, the single network hydrogel prepared by cross-linking sodium alginate (SA) oxide and chitosan, relies on Schiff base bonding alone and is associated with poor mechanical and glue-forming properties; hence, it requires modification and optimization to enhance its properties. Currently, there are various means used to enhance the mechanical properties of hydrogels, such as metal ion-based and polymer nanocomposite hydrogels. Numerous studies have shown that the incorporation of nanomaterials provides a good mechanical enhancement to polymeric materials. For example, flexible porous nanocomposite hydrogels, based on chitosan and zinc oxide nanoparticles, were prepared by Kuma et al. for wound repair. In their study, zinc oxide nanosuspension was incorporated into chitosan hydrogel and freeze-dried; the tensile strength of the resulting zinc oxide nanocomposite hydrogel was 50% higher than that of chitosan hydrogel [14]. We recently reported a self-repairing hydrogel, based on SA oxide and carboxymethyl chitosan (CMCS), incorporated with carboxymethyl-functionalized polymethyl methacrylate short nanofibers to optimize the mechanical, self-repairing, and gel properties of the hydrogel via the Schiff base and hydrogen bond interaction [15].

Another approach to enhance the mechanical properties of hydrogels is to prepare double network cross-linked hydrogels to enhance the hydrogel properties by introducing covalent or non-covalent interactions using cross-linking agents and enzymatic reactions. For instance, He et al. first prepared single mesh hydrogels using collagen polypeptide functionalized CMCS and oxidized methacrylate SA as raw materials. Then, under UV light irradiation, the mechanical properties of the formed double-network hydrogels were enhanced nearly two-fold compared to the single network by secondary cross-linking between the methacrylate groups within the hydrogels [16]. In a previous study, we prepared Schiff-base bonded hydrogels using dopamine-grafted oxidized chondroitin sulfate and carboxymethyl chitosan and modified the hydrogels under the catalytic system of horseradish peroxidase to form double cross-linked hydrogels; this double cross-linked network resulted in enhanced and improved mechanical properties of hydrogels [17]. Therefore, the design of double cross-linking was very effective in improving the mechanical strength and stability of materials. Previous studies have reported that different thiol chitosan derivatives were synthesized by amide coupling reactions, which not only improve the water-insoluble nature of chitosan but also retain the good antibacterial properties of chitosan [18]. L-cysteine contains a sulfhydryl group, which is nucleophilic and biologically active [19], and targets bacterial membranes resulting in a significant reduction in bacterial metabolism. It also has antioxidant properties. As a cross-linking agent, but-3-yn-2-one forms a reversible thiol-alkyne double-covalent additional reaction in hydrogels as a dynamic covalent bond formation reaction. Recently, studies have reported that polymer hydrogels formed by thiol-alkyne reaction displayed good mechanical properties and biocompatibility, and varied mechanical properties of the hydrogels can be adjusted by changing the concentration of the components [20]. However, there is no report of using the thiol-alkynone double addition cross-links to improve the properties of self-healing polysaccharide hydrogel materials.

Herein, we introduced small molecules of but-3-yn-2-one into the single network hydrogel formed by oxidizing alginate and cysteine-modified carboxymethyl chitosan to obtain a double network hydrogel based on Schiff and thiol-kynone reactions. We finally constructed a self-healing polysaccharide hydrogel with good mechanical properties, antibacterial ability, and biocompatibility, indicating great potential in skin tissue engineering applications.

## 2. Results and Discussion

### 2.1. Synthesis of Cysteine-Modified Carboxymethyl Chitosan/Oxidized Sodium Alginate (SH-CMCS/OSA) Hydrogel

**Figure 1:**
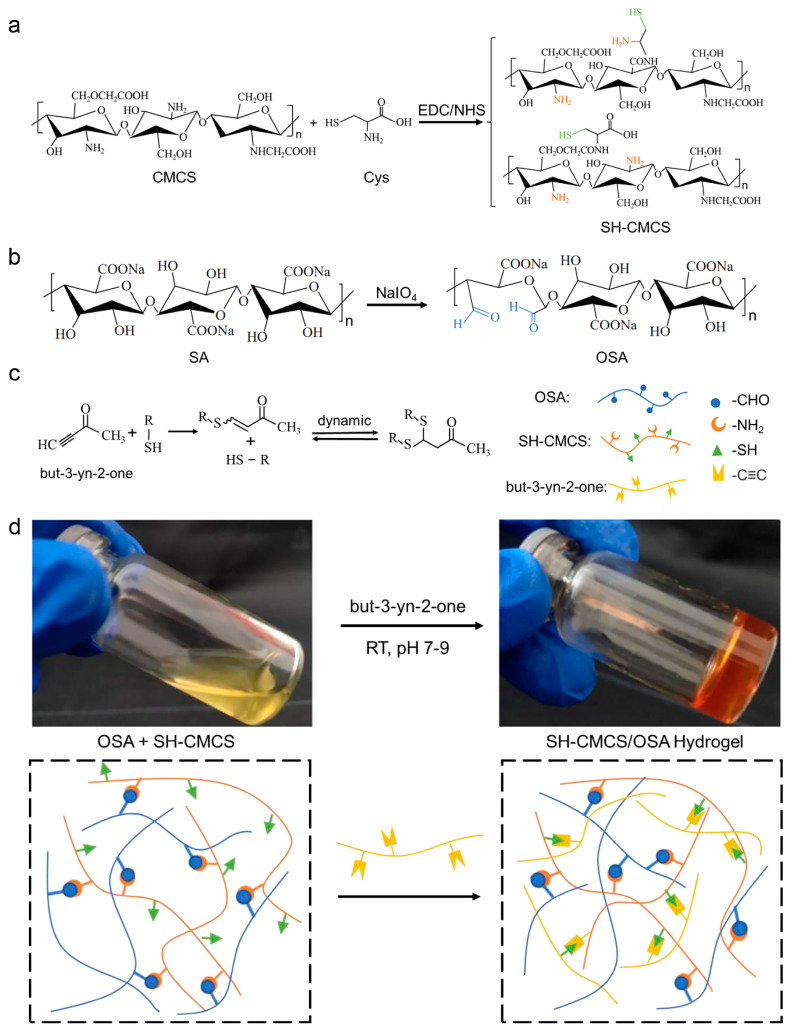
(**a**) OSA synthetic route; (**b**) CMCS graft Cys synthesis route; (**c**) Dynamic thiol-alkynone double addition; (**d**) SH-CMCS/OSA hydrogel-forming gels.

The CMCS will graft cysteine (Cys) under 1-(3-Dimethylaminopropyl)-3-ethylcarbodiimide hydrochloride (EDC) and N-Hydroxy succinimide (NHS) crosslinking to generate amide bonds, the cross-linking of carboxyl and amino groups on CMCS and Cys may produce two types of thiolated carboxymethyl chitosan as shown in Figure 1a.

Oxidized sodium alginate (OSA) was synthesized by dissolving SA in water at 70 °C and then cooling and oxidizing it with sodium periodate. Multiple functional aldehyde groups were formed on the synthesized OSA skeleton [21], and then mixed with cysteine-modified carboxymethyl chitosan (SH-CMCS) to enhance the interaction with the amino group, and the aldehyde group available on OSA can react with the Schiff base of SH-CMCS to form a colloid. The synthetic route of OSA is shown in Figure 1b.

The aldehyde group of OSA reacts with the amino group of SH-CMCS via Schiff base to form a dynamic imine bond, and when the bond is broken by an external force on the hydrogel, it can be regenerated spontaneously by the reaction between the aldehyde group and the amino group [9]. As shown in Figure 1c, in the presence of alkyne, the sulfhydryl group (-SH) of SH-CMCS undergoes an addition reaction with the carbon-carbon triple bond of alkyne [20]; the first addition between the sulfhydryl group and alkyne is an irreversible reaction, whereas the addition between the next step, i.e., the single adduct and the second sulfhydryl group, is reversible, which enhances the dynamic self-healing ability of the hydrogel. The reaction between the aldehyde group of OSA and the amino group of chitosan generates a single network hydrogel, which is not gelatinized at this time. After adding alkynyl ketone under certain conditions, a thiol-alkynyl ketone reaction occurs, and the double network gelatinized hydrogel is formed by interaction Figure 1d.

### 2.2. Characteristics of SH-CMCS/OSA Hydrogel

**Figure 2:**
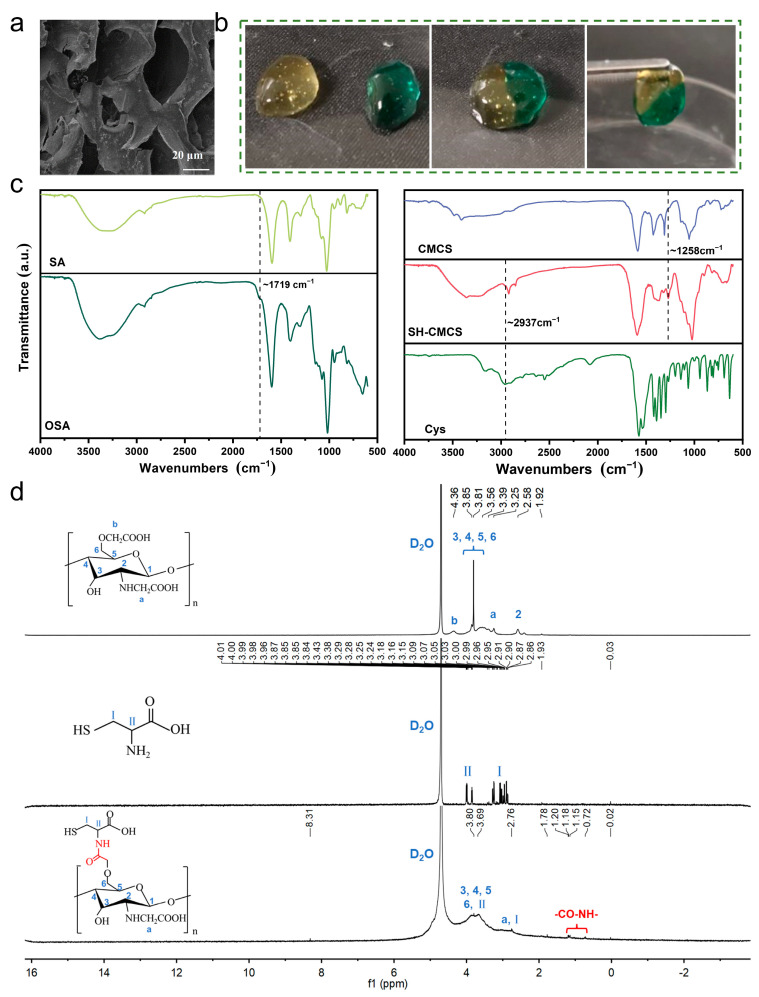
(**a**) Schematic diagram of self-healing hydrogel; (**b**) SEM diagram of SH-CMCS/OSA hydrogel; (**c**) FTIR of hydrogel; and (**d**) ^1^H NMR of CMCS, Cys and SH-CMCS.

Microscopic observation of the SH-CMCS/OSA composite hydrogel revealed that it exhibited a three-dimensional porous structure (Figure 2a), which allowed cell movement and provided an ideal environment for wound repair. Some reports have shown aromatic or aliphatic Schiff bases to be stable [22]; however, in this study, the Schiff base reaction between OSA and CMCS was not as stable as aromatic or aliphatic Schiff bases, but the addition of kynurenine gave the hydrogel a kynurenine-mercaptol double addition dynamic reversible reaction, which was also able to compensate for its stability.

To observe the self-repair process, two hydrogels were prepared, one of which was stained. The gels were cut in half and the two counter-cut halves were placed together. After 5 min, the boundary of the hydrogel splice became blurry, and 2 h later, it was able to overcome gravity and easily lifted by forceps. This demonstrated that the two different hydrogels had completed the self-repair process (Figure 2b).

The Fourier-transform infrared (FTIR) spectra of SA and OSA (Figure 2c) showed a new absorption peak at ~1719 cm^−1^ for OSA, probably due to the stretching vibration of -CHO, which also confirmed the presence of -CHO on OSA. Furthermore, we found that the absorption peak of SH-CMCS near ~2937 cm^−1^ is enhanced compared to that of Cys, possibly due to the formation of amide bond conjugation of chitosan grafted Cys, which changes the spectrum [23] These bonds are formed due to the presence of not only sulfhydryl groups but also methylene and hypomethyl groups on Cys and the characteristic peak of amide bond at ~1258 cm^−1^. These results demonstrate that we successfully grafted Cys onto CMCS by EDC/NHS crosslinking.

CMCS was functionalized with Cys to give CMCS with -SH. The proton nuclear magnetic resonance (^1^H NMR) profiles of CMCS, Cys, and CMCS-Cys in D_2_O were shown in Figure 2d. The characteristic peaks of CMCS at 2.58 ppm and in the range of 3.2–3.9 ppm corresponding to the cyclic methyl protons on CMCS [24], and the CMCS at 4.36 ppm. The characteristic peak at 4.36 ppm corresponding to the carboxymethylation of chitosan. Compared to the CMCS curve, new characteristic peaks appeared at 0.72–1.78 ppm for CMCS-Cys, which corresponded to the formation of new alkyl chains [25]. This confirms the presence of amide bonds and can demonstrate the successful grafting of Cys onto CMCS. Also, the thiol group number of SH-CMCS measured by the Ellman method was 3894 μ/g, which confirmed the thiol functionalization of CMCS.

To evaluate the ability of the hydrogels to respond to different pH, the swelling behavior of the samples in PBS (pH = 7.4) and HCl (pH = 1) was studied at 37 °C. The results showed that the hydrogel reached equilibrium swelling at around 60 min and the weight of the hydrogel no longer increased, as shown in Figure S1. Different pH media had a significant effect on the swelling ability of the hydrogel, while the hydrogel had a lower swelling rate under acidic conditions, probably due to the presence of a large number of carboxyl groups on SH-CMCS, which shifted the isoelectric point of the hydrogel towards the acidic environment, and the acidic environment had a lower swelling rate compared to neutral conditions because of the mutual attraction between the same charges [26]. While at other pH values, the repulsion between homogeneous charges is greater than the attraction, the hydrogel cross-linked network expands, and the swelling rate increases.

### 2.3. Rheological Test of Hydrogels

**Figure 3:**
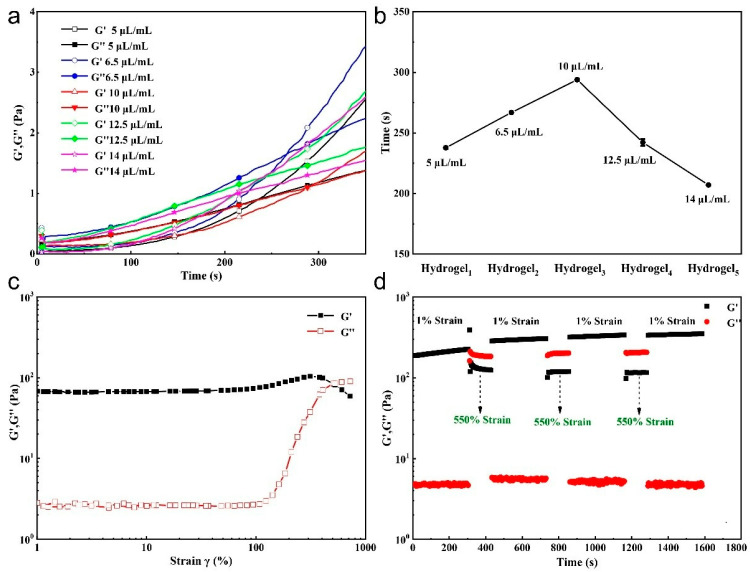
(**a**) Gelation time of SH-CMCS/OSA hydrogel at different but-3-yn-2-one concentrations; (**b**) Gelation time change of SH-CMCS/OSA hydrogel at different but-3-yn-2-one concentrations; (**c**) Maximum strain analysis of hydrogels with but-3-yn-2-one concentration of 5 μL/mL; (**d**) Rheological analysis of hydrogel at 5 μL/mL but-3-yn-2-one concentration.

The hydrogel energy storage modulus (G′) steadily increased with time, accompanied by a decrease in the loss modulus (G′′), and the intersection of these two curves was the gel point (Figure 3a). We tested the variation of gel time of SH-CMCS/OSA hydrogels at different but-3-yn-2-one concentrations (Figure 3b), and the gel time increased and then decreased with increasing but-3-yn-2-one concentration, and the minimum time required to form hydrogel was only 230 s for 5 μL/mL hydrogel solution compared with other kynurenine concentration solutions. The optimal concentration was 5 μL/mL.

When the strain was less than or equal to 100%, the G′ and G′′ of the gel remain constant, and the G′ of the hydrogel and that of the gel were greater than G′′, indicating that the hydrogel could withstand large elastic deformation. Following 100% strain, the G′ value decreased sharply and the G′′ value increased rapidly, confirming shear thinning (Figure 3c). This phenomenon indicates that the hydrogel has potentially exhibited enhanced mobility. To further characterize the mechanical properties and self-healing ability of the hydrogels, in the hydrogel rheology tests, the strains were alternately set between 1% and the maximum tolerated strain of 550%. The 1% and 550% strain durations were 130 and 120 s, respectively. When the strain was 1%, the G′ values of the hydrogels were both significantly higher than their G′′ values, indicating the structural stability of these hydrogels. However, when the strain reached 550%, G′ was lower than G′′, indicating the disrupted structure of hydrogels (Figure 3d). The hydrogels can be regenerated when the strain returns to 1% (G′ > G′′′). This alternating rheology test was performed several times, and the transition behavior between 1% and 550% strain was reproducible during the cyclic test. This indicates that the hydrogel has excellent self-healing ability and mechanical strength.

### 2.4. Cell Cytotoxicity

**Figure 4:**
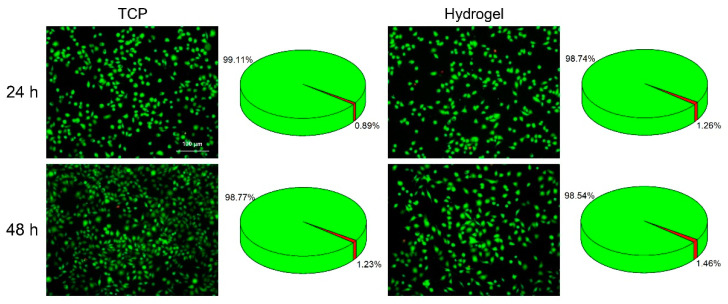
Live/dead staining of L929 cells that were treated with TCP or hydrogel for 24 h and 48 h.

Good biocompatibility is of great importance for the application of hydrogels [27]. To verify the cytocompatibility of the hydrogels, the cytotoxicity of SH-CMCS/OSA hydrogels on L929 cells (mouse fibroblasts) was evaluated using a live/dead staining method. Cells were co-incubated with the hydrogels prepared in the culture medium and cell viability was assayed after 24 and 48 h using a live/dead double staining kit. The results showed that most of the cells, in both the hydrogel and tissue culture plate (TCP) groups, appeared green in color and had a shuttle-shaped cell morphology, and the survival rate (>98%) was not significantly different at both time points (Figure 4). The results indicate that the SH-CMCS/OSA hydrogel has acceptable biocompatibility.

### 2.5. Antibacterial Properties

**Figure 5:**
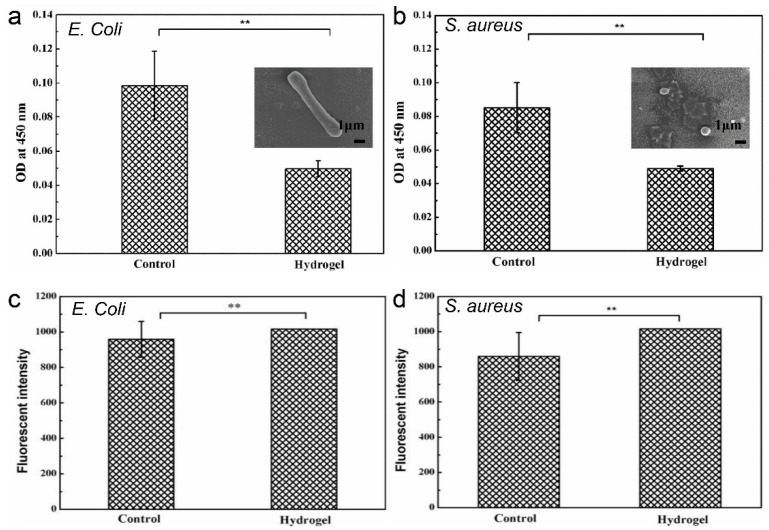
(**a**,**b**) Effect of the hydrogel on the growth of *E. coli* and *S. aureus*. (**c**,**d**) Effect of hydrogel on ROS content of *E. coli* and *S. aureus* (** *p* < 0.01).

In this study, the most commonly used organisms, *Escherichia coli* (*E. coli*) and *Staphylococcus aureus* (*S. aureus*), were selected to test the antibacterial properties of the hydrogel. Figure 5a,b showed that the hydrogel group showed a stronger antibacterial effect against both *E. coli* and *S. aureus* compared to the control group, which might have been related to the additional antibacterial activity of chitosan. The positively charged amino groups on CS can bind with negatively charged phosphopeptides or lipopolysaccharide molecules in the bacterial cell wall, destroying its integrity and rendering the organism non-viable [28]. In addition, the Schiff base complex in the hydrogel, lyses the bacteria via binding to the lipophilic layer of the bacterial cell membrane, leading to its rupturing [29,30,31]. Therefore, the composite hydrogel had a strong inhibitory effect on *E. coli* and *S. aureus*.

### 2.6. Determination of Bacterial Reactive Oxygen Species (ROS) Content

To further verify the antibacterial effect of the hydrogel on bacteria, we measured the immunofluorescence intensity in response to the ROS level in bacteria. ROS can cross the bacterial cell membrane and oxidize the mediated proteins, leading to the loss of enzyme function related to the biofilm, and the killing of the organism [32]. Figure 5c,d shows the immunofluorescence intensity measured after co-treatment of bacterial broth with hydrogel material, and the control group is the immunofluorescence intensity measured under normal bacterial growth conditions. For *E. coli* and *S. aureus*, the ROS content in the hydrogel group was significantly higher than that in the control group. We speculated that this may be due to the conversion of amino groups on chitosan to -NH^3+^, which provides enough electrons for an increased level of ROS. The composite hydrogel can utilize the positively charged amino group and ROS to achieve an antibacterial effect and inactivate bacteria.

### 2.7. In Vivo Compatibility Test

**Figure 6:**
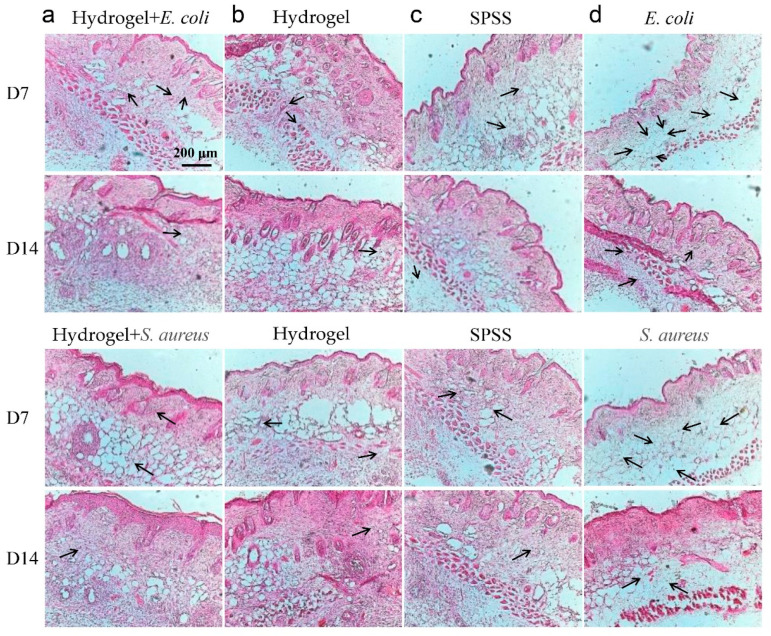
Mice skin sections stained with H&E on days 7 and 14 of (**a**) Hydrogel + bacteria; (**b**) Hydrogel-only; (**c**) SPSS; (**d**) Bacteria-only. (black arrows indicate inflammatory cells).

In order to characterize the in vivo compatibility and antibacterial properties of the hydrogel, we established four groups: Hydrogel + bacteria (*E. coli* or *S. aureus*), Hydrogel-only, saline-treated (SPSS), and Bacteria-only. Hydrogel samples were implanted under the skin of mice and inflammation was observed using hematoxylin-eosin (H&E) staining performed on days 7 and 14 after treatment. As shown in Figure 6, the hydrogel-treated group exhibited denser skin structure, compared to the control counterpart, and weaker bacterial inflammation as observed, with fewer inflammatory cell (black arrows) recruitment; a small number of hair follicles and blood vessels were also observed. However, the bacterial group exhibited severely damaged skin structure and strong inflammatory response, as indicated by the recruitment of a high number of inflammatory cells, which suggested bacteria invasion of the skin tissue.

The inflammation on day 14 indicated that the epidermis and dermis of the hydrogel group were relatively thick and there were fewer inflammatory cells compared with that on day 7. This result also reveals that the hydrogel displayed an inhibitory effect on bacteria, the skin regenerated well, and the basal layer cells proliferated and regenerated a relatively complete skin tissue structure. In contrast, poor skin regeneration was observed in the bacterial group. The inoculation of *E. coli* and *S. aureus* bacterial suspensions into the subcutaneous tissue seriously damaged the skin structure. Therefore, the employment of hydrogel can effectively prevent skin structure destruction by bacteria, accelerate the regeneration of hair follicles and blood vessels, and hasten skin tissue repair. In general, the composite hydrogel had effective antibacterial activity and good biocompatibility.

## 3. Materials and Methods

### 3.1. Materials

Carboxymethyl chitosan (CMCS; Mw: ≈100,000, DS ≥ 80%, Bomei Biotechnology, Hefei, China); Cysteine (Cys; Mw: 121.158, Qiangshun Chemical Reagent, Shanghai, China); N-Hydroxy succinimide (NHS; Rhawn, Shanghai, China); Sodium chloride (NaCl; Xilong, Guangzhou, China); Sodium alginate (SA, Mw: 198n, Sinopharm Chemical Reagent, Shanghai, China); Sodium periodate (Rhawn, Shanghai, China); Ethylene glycol (Xilong Scientific, Shantou, China); but-3-yn-2-one (Alfa Aesar, Shanghai, China); Phosphate Buffer (PB, SenBeiJia Biological Technology, Nanjing, China); Ethylenediaminetetraacetic acid disodium salt (EDTA-2Na, Xilong Chemical, Shantou, China); 5,5′-Dithiobis-(2-nitrobenzoic acid) (DTNB, aladdin, Shanghai, China); HCl (Xilong Chemical, Shantou, China); DMEM medium (Meilunbio, Dalian, China); phosphate buffered saline (PBS; SenBeiJia, Nanjing, China); Trypsin (TBD Science, Tianjin, China); Lysogeny broth medium (LB; Feiyu Bio, Nantong, China); ROS assay kit (Beyotime, Shanghai, China).

### 3.2. Synthesis of SH-CMCS and OSA

CMCS can be easily modified to meet various experimental needs, and Cys with -SH can be stabilized under acidic conditions. In this study, Cys was grafted onto CMCS to enhance its gel-forming properties. A total of 500 mg CMCS was dissolved in 50 mL distilled water, and 189 mg EDC and 114 mg NHS were also dissolved in distilled water, and then added dropwise to the CMCS solution for 10 min of low-temperature activation. A total of 100 mg Cys was completely dissolved in 50 mL distilled water and added dropwise to the previous solution, and then allowed to react for 8 h. SH-CMCS was obtained by dialyzing with 0.01 M aqueous sodium chloride solution for 3 days and then lyophilizing.

SA is a natural polymer with good biocompatibility and is widely used as a cross-linking agent. It was underutilized because of its low solubility in water. However, SA can be modified by sodium periodate oxidation to obtain aldehyde groups to enhance its water solubility. The introduction of active aldehyde groups makes it a new type of biological cross-linking agent [33]. A total of 2 g SA was completely dissolved in 100 mL distilled water at 70 °C, cooled to room temperature, and then 6 g sodium periodate was added and kept in the dark for 12 h. A total of 1 mL Ethylene glycol was added to remove excess sodium periodate and stop the reaction. After 2 h, OSA was dialyzed in cellulose membrane tubing (molecular weight cut–off of 14 kDa) against water for 3 days and freeze-dried.

### 3.3. Preparation of SH-CMCS/OSA Composite Hydrogels

The prepared SH-CMCS was dissolved in distilled water at a concentration of 20 mg/mL with sufficient stirring; the same concentration o of OSA was also dissolved in distilled water and mixed with the SH-CMCS solution to obtain SH-CMCS/OSA mixture. A total of 5 μL/mL but-3-yn-2-one was added to the mixture solution under dark conditions, and the hydrogel was formed after vigorous stirring and left to stand for 3–4 min.

### 3.4. Characteristics

The prepared hydrogels were freeze-dried, and the morphology of the hydrogels was observed by field emission scanning electron microscope (FE-SEM, ZEISS Gemini SEM 300, ZEISS, Oberkochen, Germany), and the infrared spectra of the hydrogels were analyzed by FTIR spectroscopy (TENSOR 27, Bruker, Munich, Germany) in the range of 500–4000 cm^−1^ with a resolution of 2 cm^−1^. CMCS, Cys, and SH-CMCS were dissolved in D_2_O and ^1^H NMR spectra were obtained using a fully digital NMR spectrometer (AVANCE III HD 400MHZ, Bruke, Switzerland).

The Ellman reaction was used to assay for sulfhydryl groups on SH-CMCS [34]. Briefly, 0.1 M Phosphate Buffer was prepared, 1 mM Ethylenediaminetetraacetic acid disodium salt was added to PB as the reaction buffer, and 4 mg of 5,5′-Dithiobis-(2-nitrobenzoic acid) was added to 1 mL of reaction buffer as Ellman’s reagent, 250 μL of the sample to be tested was added to 2.5 mL of reaction buffer, then 50 μL of Ellman’s reagent was added, and the reaction was carried out at room temperature for 15 min. The absorbance values were measured at 411 nm for different concentrations of cysteine solutions up to the standard curve (Figure S2) and were used to calculate the content of sulfhydryl groups.

The swelling properties of the hydrogels were investigated using different pH media. The freeze-dried hydrogel samples were immersed in PBS and 0.1 M HCl at 37 °C [34]. Three parallel samples were set up in each group and the hydrogels were removed at each time point and the excess water was blotted off with filter paper, then weighed and freeze-dried again when the weight of the hydrogel no longer increased and weighed. The swelling ratio (SR) was calculated using the following formula:

$$SR\;(\%)=\frac{\left(W_{T}-W_{s}\right)}{W_{s}}\times 100$$

*W_T_* and *W_S_* are the weight of the sample at each time point and the weight of the final freeze-dried sample.

### 3.5. Rheological Test of Hydrogels

Rheological properties of SH-CMCS mixed with OSA and SH-CMCS/OSA composite hydrogels were evaluated and compared after the addition of kynurenine using a Haake MARS rotational rheometer (Thermo Fisher Scientific, Waltham, MA, USA). Three sets of parallel samples were set up for each system.

### 3.6. Cell Cytotoxicity

The freeze-dried SH-CMCS/OSA hydrogel samples were sterilized and mixed proportionally with DMEM medium and added to 24-well plates. L929 cells were washed with PBS, digested in trypsin, and centrifuged. Resuspended cells were added to the wells at 1 × 10^4^ cells/well and incubated at 37 °C and 5% CO_2_ for 24 and 48 h. The cells were then incubated at time points with 50 μL of calcein AM and propidium iodide (PI) live/dead cell double staining kit (Beyotime, Shanghai, China) to determine cell viability, incubated at 37 °C in the dark for 30 min. Images were acquired using an inverted fluorescent microscope (Ti-DH, Nikon, Tokyo, Japan).

### 3.7. Antibacterial Properties

The hydrogel samples were processed into matching circles and sterilized overnight under ultraviolet light. *E. coli and S. aureus* strains were grown in 5 mL lysogeny broth medium (5 mL) at 37 °C in a shaker (200 rpm) incubator overnight. Aliquots (10 μL) (1 × 10^5^ CFU/mL) were inoculated into fresh LB medium. Then, the sterilized hydrogel samples were added to the culture media and incubated at 37 °C overnight. The following day, the absorbance (OD_450nm_) of the solutions was measured using a multi-labeled microplate detector (Enspire 2300, PerkinElmer, CA, USA). The experiment was performed in triplicate.

### 3.8. ROS Analysis

Intracellular ROS production levels were detected using 2′,7′-dichlorofluorescein diacetate (DCFH-DA). *E. coli* and *S. aureus* were co-incubated with SH-CMCS/OSA composite hydrogel for 4 h. Untreated pure cultures of the bacteria acted as control groups. Using the ROS assay kit, 10 μL of probe-loaded bacteria were added to each of the culture solutions. After irradiation with a 660 nm LED lamp for 10 min, the data were analyzed employing a fluorescence spectrophotometer (RF-5301PC, Shimadzu Co., Ltd., Kyoto, Japan) at an excitation wavelength of 488 nm and emission wavelength of 525 nm.

### 3.9. In Vivo Compatibility Test

After UV sterilization of SH-CMCS/OSA overnight, the hydrogel samples were processed into identical circles to maintain sample consistency in the experiments. All procedures performed on experimental animals were approved by the Animal Ethics Committee of Nantong University. The control groups were treated with saline and *E. coli* and *S. aureus* suspensions. Female mice (6) were anesthetized, and a 1 cm-length skin wound was executed on the back of the mice. The hydrogel was implanted into the subcutaneous tissue, and the mice were sacrificed on days 7 and 14 and skin tissue was then removed. The skin tissue samples were embedded and sliced into 8-μm thick sections. The tissue sections were stained using H&E staining, sealed with neutral gum, and observed under a microscope.

## 4. Conclusions

In this study, chitosan grafted with Cys was prepared and mixed with OSA in the presence of acetyl ketone to form a gel, and SH-CMCS/OSA composite hydrogel materials were successfully prepared. We evaluated the morphology, self-healing properties, and rheological properties of the composite hydrogel and found that the addition of but-3-yn-2-one provided more stable hydrogel properties and enhanced the self-healing ability of the composite hydrogel by dynamic ketone-mercapto alcohol addiction reaction. The results indicated that SH-CMCS/OSA composite hydrogel not only possessed good biocompatibility but also had strong mechanical properties. Moreover, the SH-CMCS/OSA composite hydrogel had antibacterial activity, which is further verified by ROS detection. The in vivo compatibility test proved that SH-CMCS/OSA composite hydrogel has antibacterial activity, and in vivo biocompatibility, and can inhibit inflammation and accelerate the repair of wound skin tissue. Therefore, the SH-CMCS/OSA composite hydrogel has excellent physicochemical properties and in vivo biocompatibility, providing a new and efficient wound dressing that broadens the choices of wound dressings and opens up possibilities for further research into skin tissue engineering, skin tissue repair mechanisms, and preventing wound infections.

## Data Availability

All relevant data are presented in the manuscript; raw data are available upon request from the corresponding authors.

## Supplementary Materials

The following supporting information can be downloaded at: https://www.mdpi.com/article/10.3390/ijms232213817/s1.
