# Supplementary material for: Dynamic Double Cross-Linked Self-Healing Polysaccharide Hydrogel Wound Dressing Based on Schiff Base and Thiol-Alkynone Reactions
Source: Int J Mol Sci. 2022 Nov 10;23(22):13817. doi: 10.3390/ijms232213817 (PMC9699423; doi:10.3390/ijms232213817)
Supplement: Supplementary file 1 [file ijms-23-13817-s001.zip › ijms-1993248-supplementary.pdf]

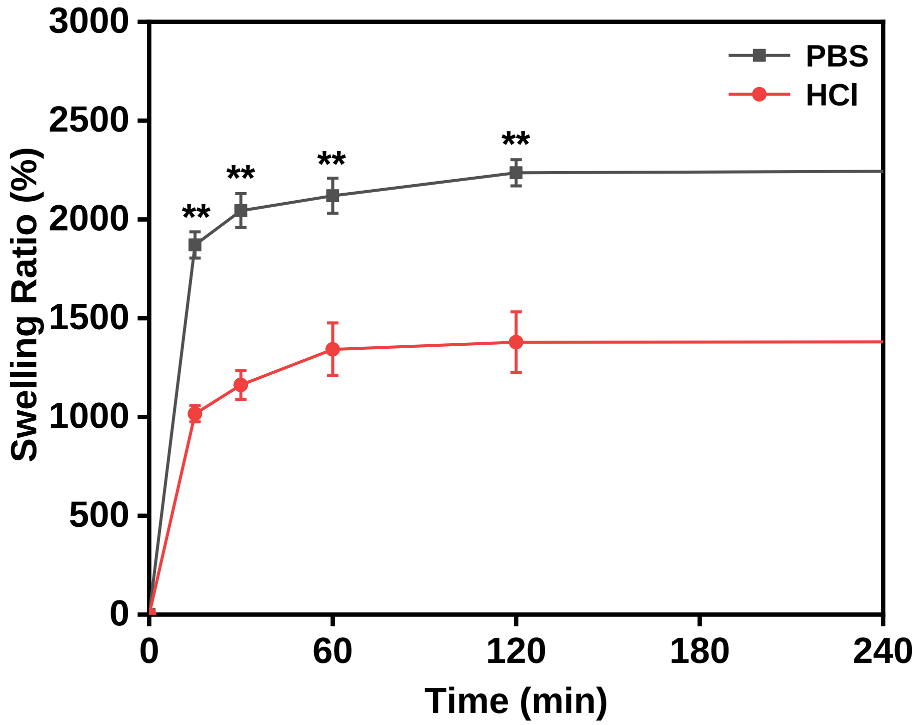

Figure S1. Swelling ratio of SH-CMCS/OSA hydrogel in PBS and HCl at 37°C (**$p < 0.01$).

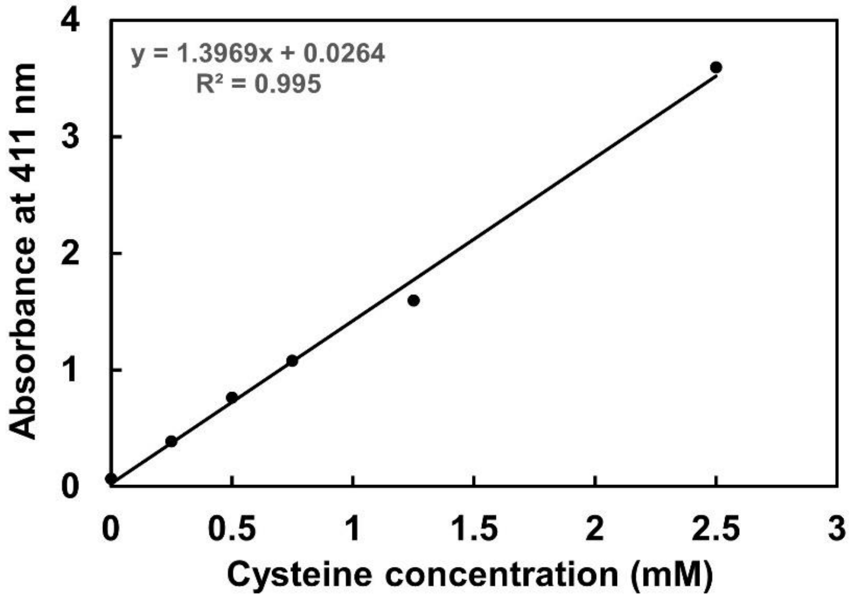

Figure S2. Cysteine standard curve.
